# Neferine suppresses autophagy‐induced inflammation, oxidative stress and adipocyte differentiation in Graves' orbitopathy

**DOI:** 10.1111/jcmm.15931

**Published:** 2021-01-14

**Authors:** Hong Li, Long Gao, Jie Min, Yucheng Yang, Ren Zhang

**Affiliations:** ^1^ Department of Endocrinology Shanghai University of Traditional Chinese Medicine Longhua affiliated Hospital Shanghai China

**Keywords:** adipocyte differentiation, autophagy, Graves’ orbitopathy, inflammation, Neferine, oxidative stress

## Abstract

Previous studies in Graves’ orbitopathy (GO) patient‐derived fibroblasts showed that inhibition of autophagy suppresses adipogenic differentiation. Autophagy activation is associated with inflammation, production of reactive oxygen species and fibrosis. Neferine is an alkaloid extracted from *Nelumbo nucifera,* which induces Nrf2 expression and inhibits autophagy. Here, we have elucidated the role of neferine on interleukin (IL)‐13‐induced autophagy using patient‐derived orbital fibroblasts as an in vitro model of GO. GO patient‐derived orbital fibroblasts were isolated and cultured to generate an in vitro model of GO. Autophagy was determined by Western blot detection of the markers such as Beclin‐1, Atg‐5 and LC3 and by immunofluorescence detection of autophagosome formation. Analysis of differentiation towards an adipogenic lineage was performed by Oil red O staining. The expression of inflammatory factors was detected by ELISA and semiquantitative RT‐PCR. Neferine inhibited autophagy in GO orbital fibroblasts, as indicated by the suppression of IL‐13‐induced autophagosome formation, overexpression of autophagy markers, increased LC3‐II/LC3‐I levels and finally down‐regulation of p62. Neferine suppressed IL‐13‐induced inflammation, ROS generation, fibrosis and adipogenic differentiation in GO patient‐derived orbital fibroblasts. The anti‐inflammatory, antioxidant and antiadipogenic effects of neferine were accompanied by the up‐regulation of Nrf2. These results indicated that orbital tissue remodelling and inflammation in GO may be mediated by autophagy, and neferine suppressed autophagy‐related inflammation and adipogenesis through a mechanism involving Nrf2.

## 1 INTRODUCTION

Graves’ orbitopathy (GO) is an autoimmune disorder with an occurrence in approximately 50% of patients with Graves’ disease. In the follicular cells of the thyroid gland, autoantibodies binds to the thyrotropin receptor, leading to the excessive production of thyroid hormones in GO.^1^, ^2^ It is reported that significant serum IL‐13 increases (*P* < .05) were found in GO patients compared with healthy controls.^3^ Moreover, IL‐13 gene polymorphisms are associated with Graves' disease (GD) susceptibility in Japan.^4^ GO is characterized by the retraction of the upper eyelid, periorbital tissue erythema, proptosis and oedema caused by an increase in orbital connective tissue and an overabundance of adipose tissue within the bony orbit.^5^, ^6^ In GO, orbital adipose tissue is characterized by the abundance of pre‐adipocytes and adipocyte differentiation is a known feature of GO; however, the underlying mechanisms remain unclear.

The key characteristics of differentiation from pre‐adipocytes to adipocytes is lipid droplets formation in the cytosol, and also, the process of autophagy is strongly linked with adipose tissue formation.^7^ Autophagy in a cell is a controlled degradation system by which cytoplasmic components are enveloped into structures, which are double membraned known as autophagosomes and targeted for lysosomal degradation. Autophagy involves the p62/sequestosome, an autophagic adapter with interacts with the microtubule‐associated protein light chain 3 (LC3; autophagosomal marker).^8^ LC3 is present in two forms: LC3‐I and LC3‐II; LC3‐II is created from LC3‐I by its conjugation with phosphatidylethanolamine, which is drawn into autophagosomal membranes and degraded by lysosome.^9^ Therefore, the ratio of LC3‐II to LC3‐I is a marker of autophagy.

The p62 gene is induced by oxidative stress through nuclear factor erythroid 2‐related factor 2 (Nrf2), a transcriptional factor which protects the cells against oxidative stress‐induced damage.^10^ Kelch‐like ECH‐associated protein 1 (Keap‐1) binds and negatively regulates Nrf2 in the cytoplasm and targets it for proteasomal degradation.^11^, ^12^ The Keap1‐Nrf2 complex is disrupted in by response to oxidants and this further leads to the stabilization and nuclear translocation of Nrf2, where it further regulates the expression of antioxidant and anti‐inflammatory genes. Therefore, alterations in Nrf2 signalling increase oxidative stress‐induced inflammation.

Neferine, an extract from the traditional Chinese medicinal plant *Nelumbo nucifera,* has been identified to induce autophagy via inhibition of PI3K/Akt signalling and the generation of reactive oxygen species (ROS).^13^ However, neferine was also shown to inhibit autophagy by activating Akt/mTor signalling and promoting the nuclear translocation of Nrf2.^14^ In the current study, we examine the therapeutic effects of neferine in a model of interleukin (IL)‐13‐induced autophagy in primary GO patient‐derived orbital fibroblasts and explored the underlying mechanisms.

## 2 MATERIALS AND METHODS

### 2.1 Reagents and chemicals

**FIGURE 1:**
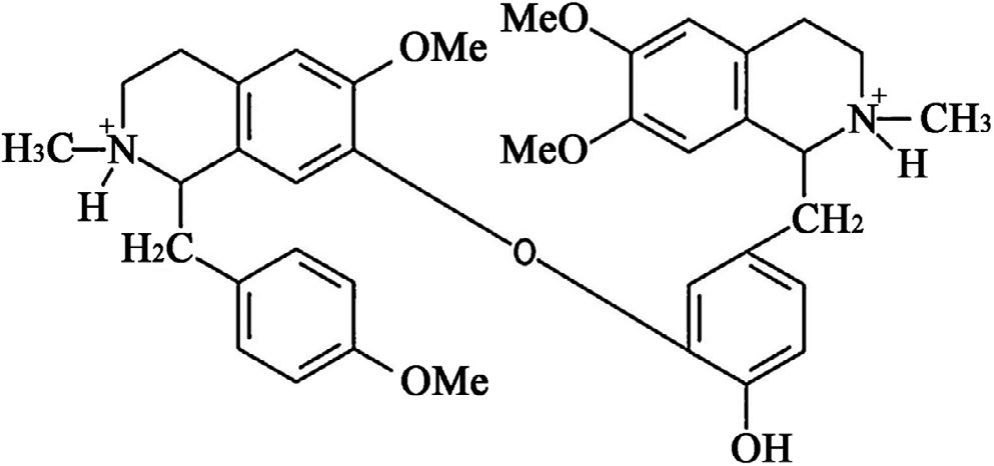
Structure of neferine

High glucose Dulbecco's modified Eagle's medium (DMEM), foetal bovine serum (FBS) and adipogenic differentiation media (A1007001) was supplied by GIBCO(USA), gentamicin solution and penicillin‐streptomycin solution was purchased from Sangon Biotech (Shanghai China), and GFP‐LC3 labelled plasmids were purchased from Yuanke (Shanghai, China); 3‐Methyladenine (3‐MA) and TRIzol were purchased from Gibco‐BRL (USA). ELISA kits were obtained from Promega (USA). All antibodies were obtained from Santa Cruz Biotechnology, Inc (USA). Neferine was purchased from MedChemExpress(USA). The structure of neferine is shown in Figure 1. Other laboratory reagents were purchased from Sigma.

### 2.2 Orbital fibroblast cell culture

The isolation and extraction of patient‐derived fibroblast was performed as mentioned previously.^15^ Monolayer cells were passaged gently with trypsin/EDTA, and the cells used for the experiments were usually from the third and fifth passages. Approvals for experiments were provided by the Longhua Hospital Ethics Committee of China, and appropriate informed consent was provided by the patients. This study was performed with respect to the tenets of the Declaration of Helsinki. 3‐MA is a widely used inhibitor of autophagy via its inhibitory effect on PI3K,^16^ which may be involved in IL‐13–mediated inflammation in mice.^17^ Initially, the GO patient‐derived fibroblasts were pre‐treated with 3‐MA or Neferine in DMEM medium supplemented with 1% FBS and further the cells were treated with IL‐13.

### 2.3 Western blot analysis

10% SDS‐PAGE gels were used to migrate the cell lysates which was further transferred to Hybond ECL nitrocellulose membranes. Successful blocking of the membranes was achieved by incubation with TBS buffer for 12 hours. Primary antibodies such as Beclin‐1, Atg‐5, LC3 and GAPDH were incubated with the blot at 4°C overnight. Subsequently, washing was performed to remove unbound antibodies and the membrane was incubated with horseradish peroxidase‐conjugated secondary antibody for 1 hour at room temperature. Using chemiluminescence reagents, protein bands were detected (Invitrogen, USA), and further quantification was done by densitometric analysis using ImageJ software.

### 2.4 Enzyme‐linked immunosorbent assay (ELISA)

IL‐6 and 8, TNF‐α and monocyte chemoattractant protein‐1 (MCP‐1) concentrations were detected using ELISA kits in cell culture supernatants and mouse serum samples following the manufacturer's instructions.

### 2.5 Immunofluorescence

Immunofluorescence‐based staining was used to detect autophagic membrane marker LC3. Cells were cultured on glass coverslips and either treated with/without 3‐MA (5 μM) or neferine (500 nM) with IL‐13 (10 ng/mL) induction for 7 days. Cells were further fixed for 15 minutes with 4% paraformaldehyde. Cells were permeabilized with 0.1% Triton X‐100 for 10 minutes, and 3% bovine serum albumin in PBS was used to block the cells for 30 minutes. Further, the cells were incubated with anti‐LC3 antibodies overnight, which was followed by an incubation with conjugated secondary antibody for 1 hour at room temperature in the dark. After several washes with PBS, the slides were incubated with DAPI for 3 minutes. The slides were mounted with glycerol, and fluorescence was visualized with a fluorescence microscope.

### 2.6 Measurement of intracellular ROS

Carboxy‐H_2_DFFDA was used to detect ROS generation wherein cells were treated with serial concentrations of IL‐13 (10 ng/mL) with/without 3‐MA (5 μM) or neferine (500 nM), and the cells were incubated at 37°C in 5% CO_2_ for 7 days. The cells were washed with DPBS after 7 days and were incubated with 10 μM Carboxy‐H_2_DFFDA for 30 minutes, and the cells were further trypsinized and washed in cold DPBS. Flow cytometry was used to measure the fluorescence intensity (FACSverse; BD Biosciences, Franklin Lakes, NJ, USA), and Flow JO software was used to perform the analysis (TreeStar, Inc, Ashland, OR, USA).

### 2.7 Autophagy assay

Autophagy was detected through the autophagy marker LC3, cells were transfected with GFP‐LC3 plasmids according to the manufacture and the fluorescence was detected during autophagosome formation through fluorescence microscopy as described previously.^18^, ^19^

### 2.8 Adipocyte differentiation assay

To determine the effect of autophagy on adipocyte differentiation, orbital fibroblasts were cultured in adipogenic differentiation medium which contains adipogenesis supplements required for adipocyte differentiation, for 2 weeks with or without pre‐treatment with 3‐MA (5 μM) or neferine (500 nM) under IL‐13 induction condition. Adipogenic differentiated cells were stained with Oil red O. A light microscope was used to identify fat droplets and calcium deposits.

### 2.9 RNA isolation and semiquantitative RT‐PCR

TRIzol method was used to isolate total RNA was from various pre‐treated GO orbital fibroblasts according to the manufacturer's instructions (Invitrogen, Carlsbad, CA). Reverse transcriptase was performed by utilizing equal amounts of RNA along with RT mix (Thermo Scientific) and oligo‐dT as a primer. The resulting templates were subjected to PCR using the following specific primers: GAPDH (sense 5′‐CATCTTCTCAAAATTCGAGTGACAA‐3′, antisense 5′‐AGTAGACTCCACGACATACTCA‐3′); TNF‐α (sense 5′‐ACTGCTCTGGAGAGCAAACA‐3′, antisense 5′‐GTGGTACATCGAGTGCAGCC‐3′); IL‐1β (sense 5′‐CAGATGAAGTGCTCCTTCCA‐3′, antisense 5′‐CATAAGCCTCGTTATCCCAT‐3′); IL‐6 (sense 5′‐CTAGATGCAATAACCACCCC‐3′, antisense 5′‐CCGAAGAGCCCTCAGGCTGG‐3′); MCP‐1 (sense 5′‐GCTGCTCATAGCAGCCACCT‐3′, antisense 5′‐AGGTGGTCCATGGAATCCTG‐3′).

### 2.10 Statistical analysis

All data are presented as the mean ± SD. GraphPad Prism 6.0 software was used to perform the data analysis. Student's *t* test was used to analyse the difference between two groups which were analysed. One‐way analysis of variance (ANOVA) followed by Tukey's multiple comparisons test was used to perform multiple group comparison. Values with a *P* value < .05 were considered statistically significant.

## 3 RESULTS

### 3.1 IL‐13 treatment induces autophagy in GO patient‐derived orbital fibroblasts

**FIGURE 2:**
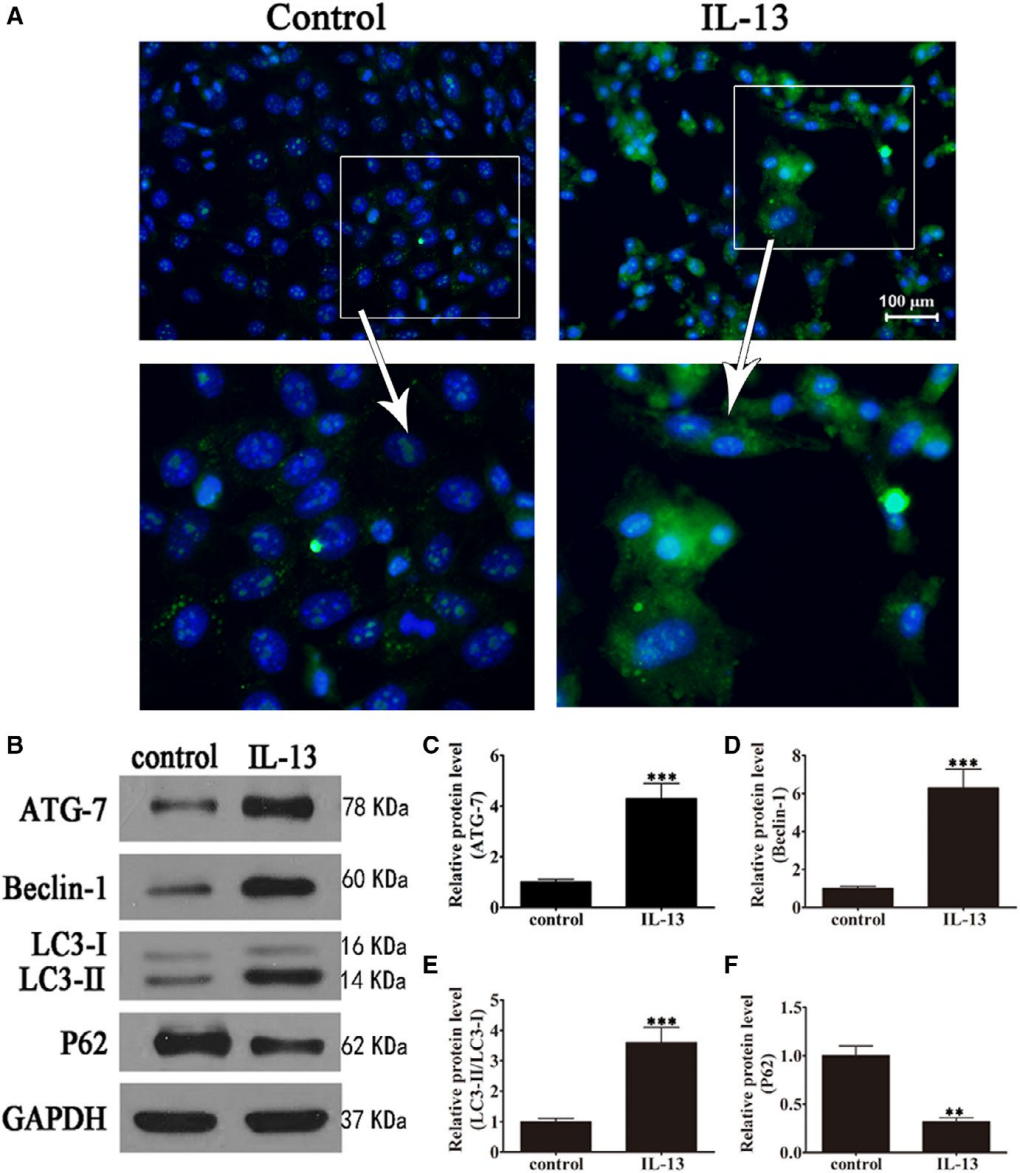
IL‐13 induction promoted autophagy in GO orbital fibroblasts. GO orbital fibroblasts were treated with or without IL‐13 (10 ng/mL) for 7 days. (A) Representative immunofluorescence images of cells transfected with GFP‐LC3 (magnification: 40×). (B–F) Western blot analysis of the expression of the autophagy‐related proteins Atg‐7, Beclin‐1, LC3I/II and p62 and densitometric quantification of bands. GAPDH was used as the loading control. Data indicate the mean ± SD (n = 5). **P < .01, ***P < .001 vs control

GO patient‐derived orbital fibroblasts were treated with IL‐13, and the induction of autophagy was examined by immunofluorescence and Western blotting. Figure 2A shows representative images of cells transfected with GFP‐LC3, illustrating autophagosome formation induced by IL‐13. IL‐13 significantly up‐regulated Atg‐7 and Beclin‐1 whereas significantly increased the LC3‐II/LC3‐I ratio and down‐regulated p62 in GO orbital fibroblasts, indicating the induction of autophagy (Figure 2B–F).

### 3.2 IL‐13‐induced autophagy promotes inflammation, ROS generation, and fibrosis and stimulates adipocyte differentiation

**FIGURE 3:**
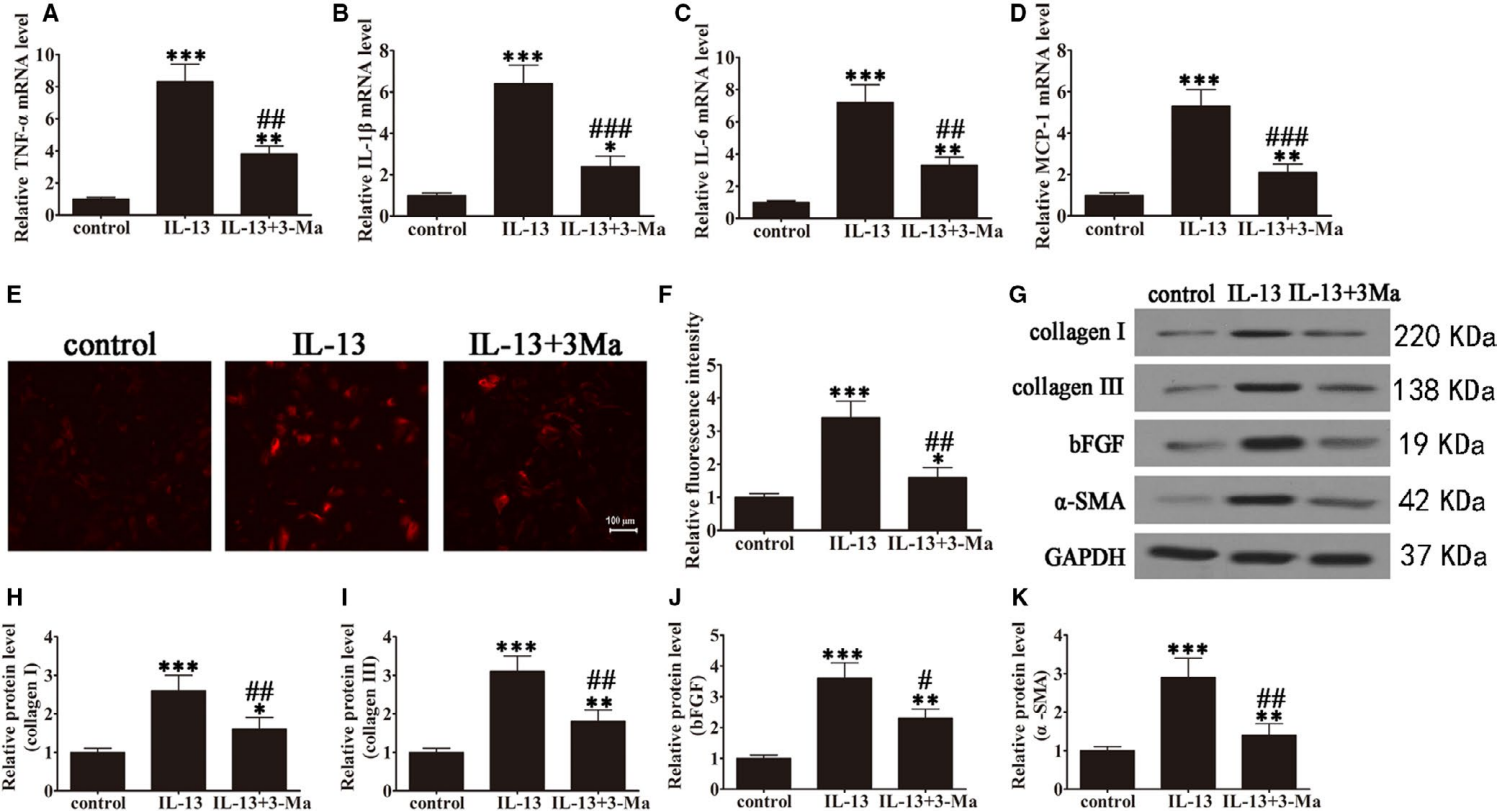
IL‐13 induced autophagy‐promoted inflammation, ROS production and fibrosis in GO orbital fibroblasts. GO orbital fibroblasts were treated with or without IL‐13 (10 ng/mL) in the presence or absence of 3‐methyladenine (3‐MA, 5 μM) for 7 days. Untreated cells were included as controls. (A–D) The mRNA levels of TNF‐α (A), IL‐1β (B), IL‐6 (C) and MCP‐1 (D) were determined by RT‐PCR. Data were normalized to GAPDH. n = 5; *P < .05, **P < .01, ***P < .001 vs control; ^##^ *P* < .01, ^###^ *P* < .001 vs IL‐13. (E and F) Micrographs of GO orbital fibroblasts in the different treatment groups were labelled with DCFH to detect ROS (magnification: 40×) and ROS levels were quantified. Data are presented as the mean ± SD. n = 10; *P < .05, ***P < .001 vs control. ^##^ *P* < .01 vs IL‐13. (G–K) Western blot analysis and densitometric quantification of the expression of collagen I, collagen III, basic fibroblast growth factor (bFGF) and α‐smooth muscle actin (α‐SMA). Values are presented as the mean ± SEM (n = 5), *P < .05, **P < .01, ***P < .001 vs control; ^#^ *P* < .05, ^##^ *P* < .01 vs IL‐13

**FIGURE 4:**
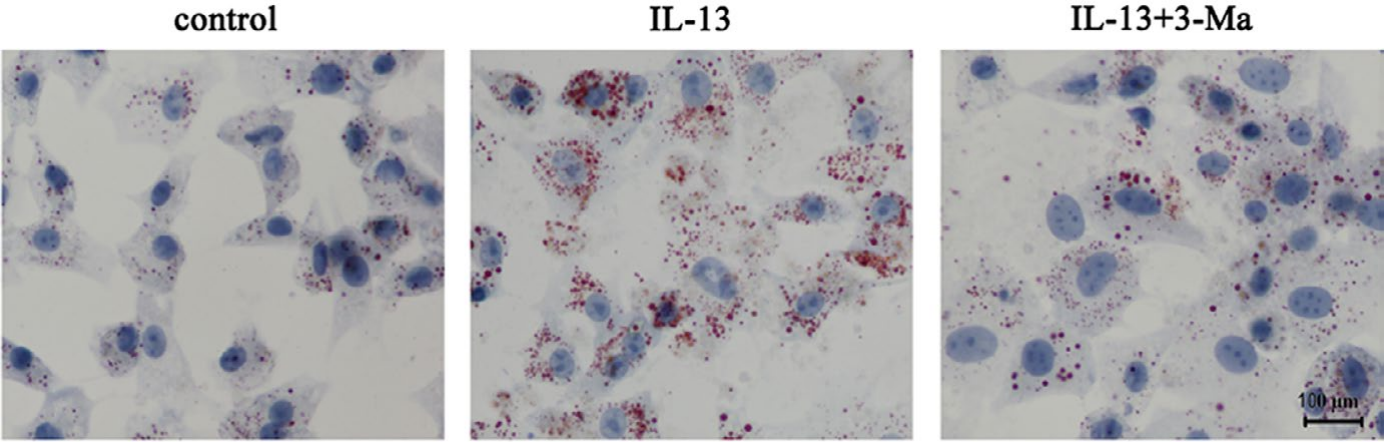
IL‐13 induced autophagy‐promoted adipocyte differentiation in GO orbital fibroblasts. GO orbital fibroblasts treated with or without IL‐13 were incubated in adipocyte differentiation medium for 2 weeks in the presence or absence of 5 μM 3‐MA. Oil Red O staining shows the effect of IL‐13 on lipid droplet accumulation

To examine the effect of IL‐13‐induced autophagy on inflammation, ROS production and fibrosis, GO patient‐derived orbital fibroblasts were initially treated with or without IL‐13 and with or without the 3‐MA (autophagy inhibitor). The results indicated that IL‐13 treatment significantly up‐regulated TNF‐α, IL‐1β, IL‐6 and MCP‐1, but these effects could be partially restored by 3‐MA, indicating that inflammation was associated with autophagy induction in GO orbital fibroblasts (Figure 3A–D). Carboxy‐H2DFFDA labelling and quantification of fluorescence showed that IL‐13 increased ROS production significantly, and 3‐MA treatment restored ROS levels (Figure 3E and F). Western blot detection of fibrosis‐associated proteins showed that IL‐13 significantly up‐regulated collagen I, collagen III, bFGF and α‐SMA in GO orbital fibroblasts, whereas 3‐MA partially reversed this effect, indicating that fibrosis was associated with the activation of autophagy (Figure 3G–K). Oil red O staining showed that autophagy inhibition by 3‐MA suppressed IL‐13‐induced oil droplet accumulation, suggesting that adipogenesis involved the induction of autophagy in primary GO orbital fibroblast cultures (Figure 4).

### 3.3 Neferine suppresses IL‐13 induced autophagy by up‐regulating Nrf2

**FIGURE 5:**
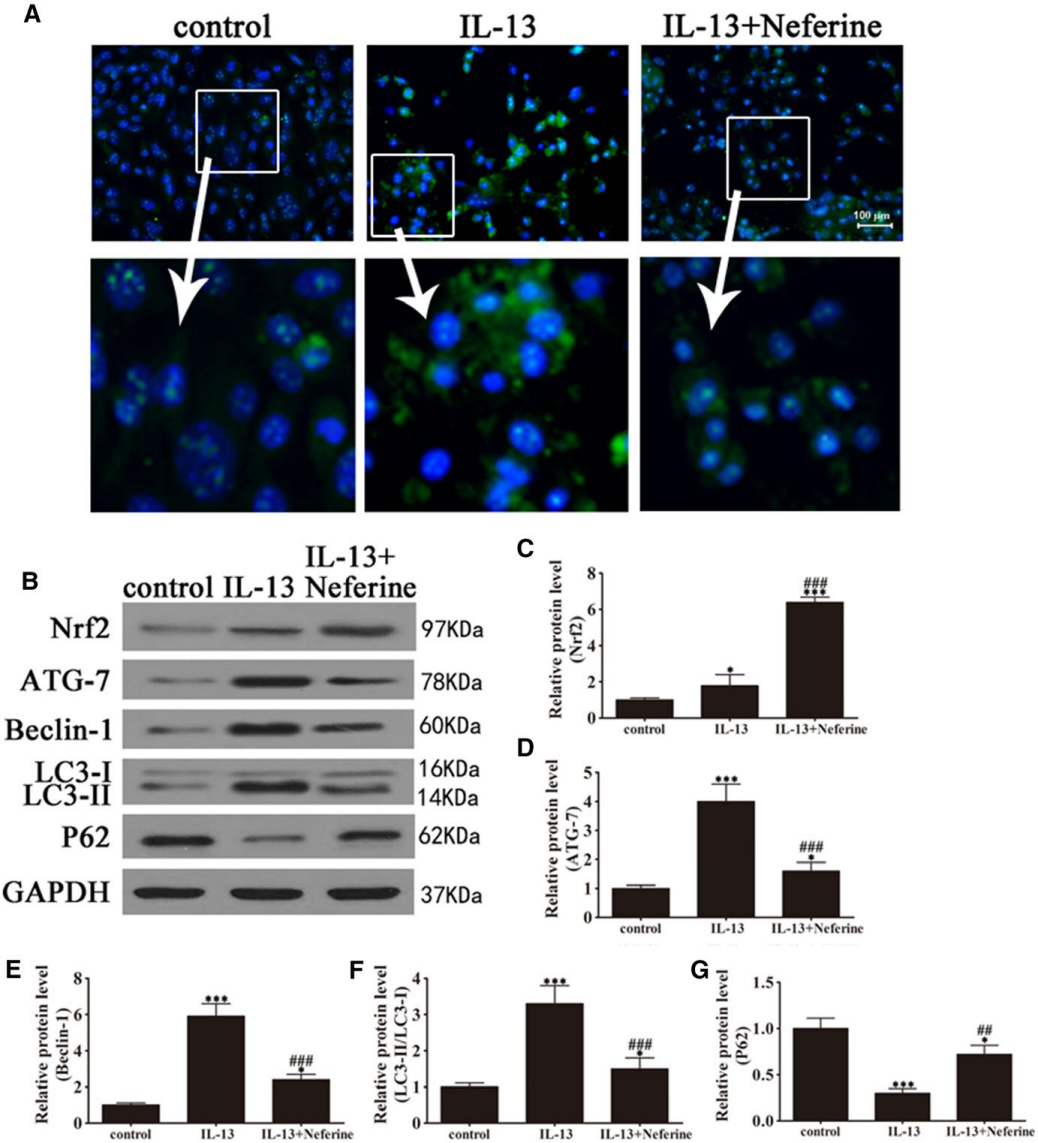
Neferine inhibited IL‐13‐induced autophagy activation by up‐regulating Nrf2 expression in GO orbital fibroblasts. GO orbital fibroblasts were treated with IL‐13 (10 ng/mL) in the presence or absence of neferine (500 nM) for 7 days. (A) Representative immunofluorescence images of LC3 puncta (magnification: 40×). (B–G) Western blot analysis and densitometric quantification of the expression of autophagy‐related proteins. Data indicate the mean ± SD (n = 5). *P < .05, ***P < .001 vs control; ^##^ *P* < .01, ^###^ *P* < .001 vs IL‐13

Next, the effect of neferine on autophagy and inflammation in GO orbital fibroblasts were assessed. Cells were treated either with or without IL‐13 in the presence or absence of neferine. Based on the immunofluorescence analysis of GFP‐LC3 transfected cells, it was evident that neferine suppressed the formation of autophagosomes after 7 days of treatment (Figure 5A). Western blotting analysis and density‐based quantification of bands indicated that neferine significantly suppressed the IL‐13‐associated up‐regulation of Atg‐7 and Beclin‐1, the increase in the LC3‐II/LC3‐I ratio, and the down‐regulation of p62, and these effects occurred in parallel with a significant up‐regulation of Nrf2 by neferine (Figure 5B–G). Hence based on the collective results, neferine might suppressed autophagy in GO orbital fibroblasts by up‐regulating Nrf2.

### 3.4 Neferine suppresses IL‐13 induced inflammation, ROS generation, fibrosis and adipocyte differentiation in GO patient‐derived orbital fibroblasts

**FIGURE 6:**
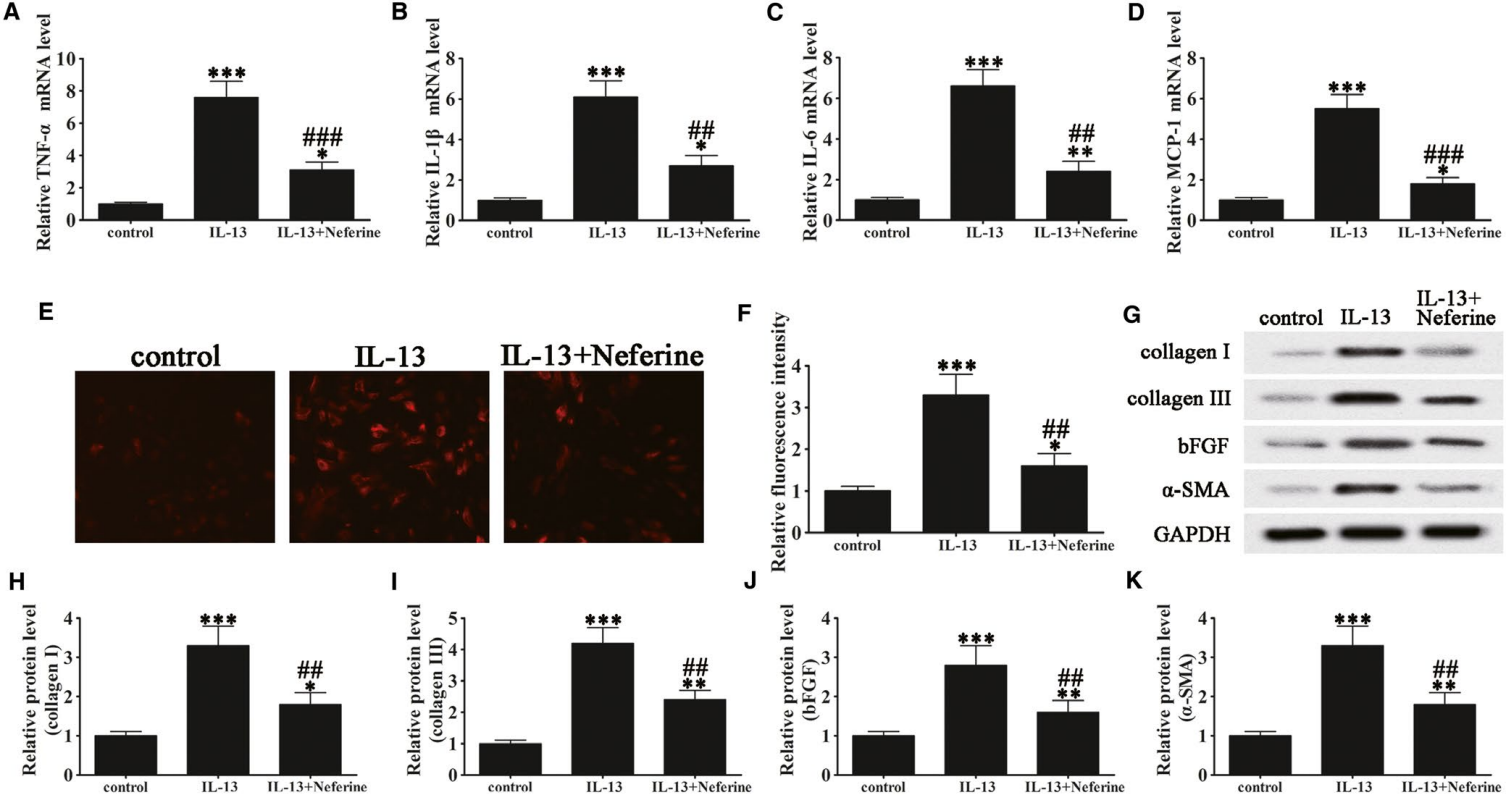
Neferine suppressed IL‐13‐induced inflammation, ROS, and fibrosis in GO orbital fibroblasts. GO orbital fibroblasts treated with or without IL‐13 (10 ng/mL) were treated with Neferine (500 nM) for 7 days. Untreated cells were included as controls. (A‐D) The mRNA levels of TNF‐α (A), IL‐1β (B), IL‐6 (C), and MCP‐1 (D) were determined by RT‐PCR. Data were normalized to GAPDH. n = 5; *P < .05, **P < .01, ***P < .001 vs control; ^##^ *P* < .01, ^###^ *P* < .001 vs IL‐13. (E and F) Micrographs of GO orbital fibroblasts labelled with DCFH to detect ROS in the different treatment groups (magnification: 40×) and quantification of ROS levels. Data are presented as the mean ± SD. n = 10; *P < .05, ***P < .001 vs control. ^##^ *P* < .01 vs IL‐13. (G–K) Western blot analysis and densitometric quantification of the expression of collagen I, collagen III, bFGF and α‐SMA. Values are presented as the mean ± SEM (n = 5), *P < .05, **P < .01, ***P < .001 vs control; ^##^ *P* < .01 vs IL‐13

**FIGURE 7:**
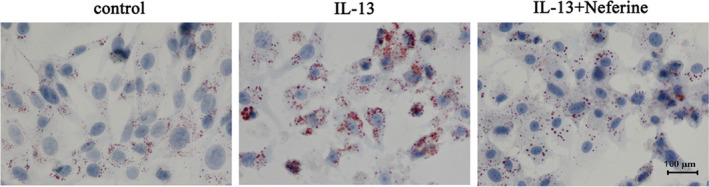
Neferine inhibited IL‐13‐induced adipocyte differentiation in GO orbital fibroblasts. GO orbital fibroblasts were incubated in adipocyte differentiation medium in the presence or absence of Neferine (500 nM) under induction of IL‐13 (10 ng/mL) for 2 weeks. Oil Red O staining showing the effect of IL‐13 on lipid droplet accumulation

The effect of neferine was assessed in GO orbital fibroblasts treated with or without IL‐13, which showed that neferine suppressed the IL‐13 induced up‐regulation of TNF‐α, IL‐1β, IL‐6 and MCP‐1 (Figure 6A–D). DCFH labelling and quantification of fluorescence showed that neferine significantly suppressed IL‐13 induced ROS generation (Figure 6E and F) and partially restored the IL‐13 up‐regulation of collagen I, collagen III, bFGF and α‐SMA (Figure 6G–K). Furthermore, neferine partially suppressed IL‐13 induced adipogenesis, as determined by Oil red O staining of orbital fibroblasts (Figure 7). Hence, these results further showed that neferine might protect cells against autophagy‐induced inflammation and adipocyte differentiation.

## 4 DISCUSSION

The symptoms of GO, particularly the increase in the of orbital connective tissue and fatty tissue volume, are accompanied by the infiltration of immunocompetent cells and proinflammatory cytokine production resulting in adipogenesis.^6^, ^20^ Although oxidative stress has been suggested as a possible cause of adipogenesis and fibrosis, the underlying specific mechanisms remain unclear, and there is currently no effective treatment for GO. In the present study, autophagy was induced in orbital fibroblasts, and the effects of neferine on autophagy‐related inflammation, oxidative stress and fibrosis were examined. We showed that neferine might suppress autophagy‐induced inflammation and adipogenesis and that these effects may be mediated by the up‐regulation of Nrf2.

In the present study, IL‐13 induced autophagy in GO orbital fibroblasts promoted inflammation, as determined by the up‐regulation of proinflammatory factors, and neferine treatment suppressed autophagy‐induced inflammatory responses. Autophagy, a protective mechanism that regulates homeostasis in eukaryotic organisms, acts via crosstalk with immunity and inflammation pathways, and defects in autophagy therefore underlie the pathogenesis of many inflammatory diseases.^18^ The early stages of GO are characterized by the interaction of infiltrating T cells with orbital fibroblasts, which further leads to the production of cytokines and T‐cell activating factors secretion.^6^ Therefore, the inhibition of orbital inflammation is an important therapeutic target to suppress the progression to tissue expansion and adipogenesis. Immunosuppressive drugs, such as cyclosporine, which inhibits cytotoxic T‐cell activation and the production of cytokines, have been tested for the treatment of GO, although the results are inconclusive.^21^ Glucocorticoids have also been tested because of their anti‐inflammatory effects, and intravenous methylprednisolone is effective in 70‐80% of patients; however, after drug withdrawal 10‐20% of patients face relapse.^21^, ^22^ In a previous study, quercetin, a phytoestrogen found in soya beans, vegetables and fruits, in primary cultured orbital fibroblasts was shown to reduce inflammation and adipogenesis.^20^ Similarly, the present results showed that neferine suppressed IL‐13‐induced inflammation in GO patient‐derived orbital fibroblasts through the inhibition of autophagy.

In GO, eyelid retraction and proptosis are caused by Muller's muscle inflammation and extraorbital connective tissue, and the wound healing response elicited can lead to chronic fibrosis.^23^, ^24^ Further, excessive deposition of extracellular matrix (ECM) components such as collagen and fibronectin is caused by a tissue repair mechanism associated with fibrosis.^25^ In the present study, IL‐13 induced GO orbital fibroblast fibrosis, as shown by the up‐regulation of collagen I, collagen III, bFGF and α‐SMA. Neferine treatment suppressed the effect of IL‐13 on fibrosis; however, whether neferine had a direct antifibrotic effect or its effect was mediated by the inhibition of inflammatory responses remained unclear. Cytokine infiltration can activate fibroblasts, and inhibition of inflammation can therefore inhibit fibrosis.^26^ Quercetin has antifibrotic effects in hepatic fibrosis, kidney fibroblasts and lung fibroblasts.^27^, ^28^, ^29^ Furthermore, quercetin down‐regulated fibrotic markers and suppressed matrix metalloprotease (MMP) 9 expression and activity in GO orbital fibroblasts.^20^ As MMPs play an important role in ECM remodelling, it would be of interest to examine the effect of neferine on MMP expression in orbital fibroblasts.

Recent research with the aim to understand the mechanisms behind the pathogenesis of GO highlights the role of retro‐orbital adipogenesis, and this led to the proposal of treatment strategies specifically targeting orbital tissue remodelling.^22^, ^30^ Inhibitors of the PI3K/mTOR signalling pathway decrease hyaluronan accumulation and inhibit adipogenesis in orbital pre‐adipocytes in vitro.^22^ Somatostatin analogues such as pasireotide have shown inhibitory effects on orbital adipogenesis.^20^ The results of the current study showed that neferine suppressed IL‐13 induced accumulation of lipid droplets associated with the induction of autophagy in orbital fibroblasts; therefore, further investigation of the specific effect of neferine on adipocyte differentiation is warranted.

The effects of neferine on suppressing autophagy‐induced inflammation, ROS generation, fibrosis and adipocyte differentiation in our GO model were accompanied by the up‐regulation of Nrf2. Neferine was previously shown to protect against oxidative stress by scavenging ROS and suppressing the nuclear translocation of NF‐κB.^31^ A later study by the same group showed that neferine suppresses autophagy by activating Nrf2 and the PI3K/Akt/mTOR survival pathway.^14^ These studies suggest that neferine has protective effects against oxidative stress‐induced cellular damage, which is in line with our present results. Furthermore, hypoxia, which disrupts homeostasis and is associated with cellular changes resulting in oxidative damage in relation to metabolic adaptation, promotes autophagy.^32^ Cells are protected against oxidative stress by Nrf2 through stimulation of the transcription of antioxidant genes, and Nrf2 nuclear translocation is inhibited when cells are exposed to hypoxia, which thereby inhibits antioxidant responses.^33^, ^34^ In the present study, neferine suppressed autophagy by up‐regulating Nrf2, which is consistent with previous results. Furthermore, Nrf2 down‐regulation promotes autophagy and the osteoblastic differentiation of adipose‐derived mesenchymal stem cells, suggesting that Nrf2 is a valuable target for the inhibition of autophagy and adipogenesis.^35^ The present results indicating that the protective effects of neferine are mediated by the up‐regulation of Nrf2 are of particular interest as a potential strategy for the treatment of the inflammatory, fibrotic and adipogenic effects associated with GO.

## 5 CONCLUSION

In conclusion, we showed that inflammation, ROS generation, fibrosis and adipogenesis in GO patient‐derived orbital fibroblasts are associated with the induction of autophagy, and neferine suppressed autophagy‐related effects. The anti‐inflammatory, antioxidant and antiadipogenic effects of neferine were mediated by the up‐regulation of Nrf2. In order to develop new drugs for the treatment of this disease, further detailed investigation of the specific effects of neferine on GO and the signalling pathways involved is necessary.

## CONFLICT OF INTEREST

The authors declare that they have no conflict of interest in relation to the topic of the manuscript.

## ETHICAL APPROVAL

This article does not contain any studies with human participants or animals performed by any of the authors.

## INFORMED CONSENT

Informed consent was obtained from all individual participants included in the study.

## Data Availability

All the data to support this study are included within the article.
